# Transarterial Chemoembolization of Hepatocellular Carcinoma with Idarubicin-Loaded Tandem Drug-Eluting Embolics

**DOI:** 10.3390/cancers11070987

**Published:** 2019-07-15

**Authors:** Boris Guiu, Sebastien Colombat, Lauranne Piron, Margaux Hermida, Carole Allimant, Marie-Ange Pierredon-Foulongne, Ali Belgour, Laure Escal, Christophe Cassinotto, Mathieu Boulin

**Affiliations:** 1 Department of Radiology, St-Eloi University Hospital, 34980 Montpellier, France; 2 Department of Pharmacy, Dijon University Hospital, 21000 Dijon, France

**Keywords:** hepatocellular carcinoma, HCC, chemoembolization

## Abstract

*Objective*: To describe the responses, toxicities and outcomes of HCC patients treated by transarterial chemoembolization (TACE) using idarubicin-loaded TANDEM beads. *Materials and Methods*: Seventy-two consecutive patients (mean age: 71 years (58–84 years)) with HCC were treated by TACE using idarubicin-loaded TANDEM in a first line, over a five-year period. Most patients (89%) had liver cirrhosis classified as Child–Pugh A (90%). BCLC B classification applied in 85% of cases. Baseline tumor burden was limited to one to three nodules in 92% of cases, unilobar in 88% cases, with a median tumor diameter of 55 mm (range: 13–150 mm). Toxicity was assessed using NCI CTC AE v4.0. Response was assessed using mRECIST criteria. Time-to-treatment failure (TTTF) and overall survival (OS) were also calculated based on Kaplan–Meier method. *Result*: Of 141 TACE sessions performed with bead sizes of 100 and 75 µm in 42 (29.8%) and 99 (70.2%) sessions, respectively. In 78% of all TACE sessions, the full dose of idarubicin-loaded beads was injected. Grade 3–4 AE were observed after 73 (52%) sessions, most of them being biological. Multi-organ failure was observed three days after the first TACE in a Child B patients, unfortunately leading to death. Overall, the best objective response rate (ORR) was 65%. Median follow-up lasted 14.3 months (95% CI: 11.2–18.8 months). Median TTTF and OS were 14.4 months (95% CI: 7.2–24.6 months) and 34.6 months (95% CI: 24.7—not reached) respectively. *Conclusion*: In this retrospective study involving well-selected HCC patients, high ORR and long TTTF and OS are observed after TACE using idarubicin-loaded TANDEM. A randomized trial is needed.

## 1. Introduction

Transarterial chemoembolization (TACE) is the mainstay of treatment in patients with intermediate stage hepatocellular carcinoma (HCC) [1], but it still presents considerable heterogeneity among centers and interventional radiologists (IRs) [2]. The optimal TACE regimen remains to be determined [1].

Drug-eluting embolics (DEEs) came to the market in 2006 and were intended to improve and standardize the TACE procedure, but failed to demonstrate any response or survival superiority over conventional TACE [3,4,5]. Doxorubicin is the most widely used anticancer agent for TACE [1]. Its use relies on a single-arm phase II trial from 1975 that showed some complete responses after systemic administration of doxorubicin in HCC patients [6]. However, these data have not been reproduced, and systemic doxorubicin is not used to treat HCC. Idarubicin, an anthracycline commonly used to treat leukemias, is supposed to be the most effective chemotherapeutic drug in HCC [1], based on an in vitro screening study that compared 11 drug candidates for TACE (including doxorubicin) on three HCC cell lines [7]. To our knowledge, no study has ever been performed to explore cancer cells or multi-drug resistance systems after treatment by idarubicin. TANDEM DEEs (Boston Scientific, Marlborough, Massachusetts) are a drug-delivery platform composed of a negatively charged hydrogel core that can be loaded with positively charged agents such as doxorubicin or idarubicin [8,9]. An organic polymer (Polyzene F) coating the hydrogel core creates a biocompatible outer shell. TANDEM DEEs are small and tightly calibrated with diameters ranging from 40 to 100 µm that show fast loading ability and a favorable pharmacokinetics profile with sustained release of idarubicin [9]. However, to our knowledge, very limited clinical data are available in the literature regarding idarubicin-loaded TANDEM.

The aim of the study was to report our experience of drug-eluting beads (DEBs) TACE using TANDEM DEEs and to evaluate safety, response and survival in non-resectable HCC patients.

## 2. Patients and Methods

This single-center retrospective study was performed in accordance with the Declaration of Helsinki. All study participants provided written informed consent. Our institutional review board approved the retrospective analysis of their anonymized data. This study was registered on www.clinicaltrials.gov (NCT03349957).

### 2.1. Study Population

All patients treated with idarubicin-loaded TANDEM DEEs as first-line intra-arterial treatment between September 2012 and November 2017 at St-Eloi university hospital (Montpellier, France) were assessed for inclusion in the analysis. All cases were discussed at our weekly multidisciplinary tumor board, comprising at least one interventional radiologist, one liver surgeon, one oncologist and one hepatologist. Inclusion criteria were: HCC according to histological examination or Barcelona criteria; measurable targets according to mRECIST; Child A or B7 cirrhosis (without decompensation in the past 6 months); not a candidate for surgery or ablation; age ≥ 18 years; performance status 0 or 1; platelet count ≥ 50,000/mm^3^; neutrophil count ≥ 1000/mm^3^; creatininemia ≤ 150 µmol/L; no cardiac failure.

Exclusion criteria were: follow-up < 1 month; bilio-enteric anastomosis; hepatofugal blood flow or thrombosis of the main portal trunk; segmental/lobar/main portal vein invasion; prior treatment by systemic chemotherapy or radioembolization.

### 2.2. TACE Protocol

Patients underwent TACE sessions with idarubicin-eluting embolics every 6 ± 2 weeks (i.e., on-demand regimen), depending on tumor load and response to TACE. Contrast-enhanced liver magnetic resonance imaging (MRI) evaluations were performed every 4–6 weeks after each session. MR protocol included T1- and T2-weighted imaging (axial plane) as well as dynamic contrast-enhanced (arterial-, portal-, late-phase) MR acquisition (axial plane) using gadoterate meglumine (Dotarem, Guerbet, France). Reasons for TACE discontinuation included severe TACE-related toxicity, progressive disease or decision of the patient or interventional radiologist. Two sessions were routinely scheduled in cases of bilobar disease.

Patients were assessed at baseline, during hospitalization and in consultation after each MR scan. Adverse events (AEs) and laboratory variables were investigator-graded according to the National Cancer Institute Common Terminology Criteria for Adverse Events (NCI-CTC AE) version 4.0. All patients had an MRI evaluation at baseline.

### 2.3. Interventional Treatment

TANDEM beads (100 or 75 µm; one vial containing 3 mL of beads (Boston Scientific, Marlborough, MA, USA)) were loaded with 10 mg (1 mg/mL) idarubicin (Zavedos, Pfizer, France) at the hospital pharmacy prior to TACE, according to the manufacturer’s recommendation. A 10-mg dose was selected based on the results of a dose-escalation phase I study regarding idarubicin DEB-TACE [10] and a preliminary experience and pharmacokinetics study of four patients [9]. Bead size was left at the discretion of the interventional radiologist. Just before injection, an equal volume of non-ionic contrast medium (iodixanol, Visipaque 270 (GE Healthcare S.A., Vélizy-Villacoublay, France)) was mixed in the syringe containing idarubicin-loaded TANDEM. In all cases, a 2.7F microcatheter (Progreat, Terumo, Tokyo, Japan) was used. Single-phase cone-beam CT was used to localize tumor feeders if necessary. Super-selective embolization was performed for patients with one to three nodules, while a sectorial or lobar embolization was performed for patients with greater tumor burden. Idarubicin-loaded embolics were injected slowly (≈1 mL/min), taking care to avoid reflux, until near stasis. In accordance with expert recommendations, no additional embolic material was used [11]. If stasis was achieved before the entire volume of embolic was injected, injection was discontinued.

### 2.4. Statistical Methods

Qualitative and continuous variables were described using percentages and medians with ranges (min–max) or means ± SD. Study endpoints included: tumor response using mRECIST criteria; objective response rate (ORR), defined as complete response (CR) or partial response (PR); best ORR until TACE discontinuation; disease-control rate (DCR), defined as CR or PR or stable disease; time-to-treatment failure (TTTF); overall survival (OS); and safety. TTTF was defined as the time from the first TACE session to TACE discontinuation for any reason, including disease progression, treatment toxicity, patient’s preference, or death. OS was defined as the time from the first TACE session to death (all causes). The median follow-up was evaluated using the reverse Kaplan–Meier method. TTTF and OS curves were determined using the Kaplan–Meier method. All statistical analyses were performed using STATA software version 11.0 (Statacorp, College Station, TX, USA). *p* values < 0.05 were considered significant.

## 3. Results

### 3.1. Patients and Tumors

Over the study period, 72 patients met the inclusion/exclusion criteria (Table 1). Median age was 71 years (range: 58–84 years) among 66 males and 6 females. Most patients (89%) had liver cirrhosis classified as Child–Pugh A (90%). BCLC B classification applied to 85% of cases. Five patients had been previously treated either by percutaneous ablation (*n* = 3), or by liver resection (*n* = 2). Baseline tumor burden was limited to three or fewer HCC nodules in 92% of cases, unilobar in 88% cases, with a median tumor diameter of 55 mm (range: 13–150 mm). Baseline serum AFP was 10 ng/mL (range: 3–17,660 ng/mL).

### 3.2. TACE Sessions

Overall, 141 sessions were performed among the 72 patients, of which 36 (50%), 14 (19%), 13 (18.1%), 7 (9.7%), and 2 (3%) patients received 1, 2, 3, 4 and 5 TACE sessions, respectively. Size of TANDEM beads was 100 and 75 µm in 42 (29.8%) and 99 (70.2%) TACE sessions, respectively. In 78% of all TACE sessions, the full dose of idarubicin-loaded beads was injected, whereas in the others (22%) the full dose was not injected because of early stasis. The mean total dose of idarubicin administered per session was 8.5 ± 1.9 mg.

### 3.3. Safety

Grade 3–4 AEs were observed after 73 (52%) sessions. Most of them were biological (Table 2: Grade 3–4 pain was noted in 4% of cases and successfully treated by morphin titration. No Grade 3–4 hematologic toxicity was observed. Gallbladder necrosis was observed in one case, with favorable outcome after transhepatic gallbladder drainage. Multi-organ failure was observed three days after the first TACE in a Child B patient, unfortunately leading to death.

Segmental/sub-segmental bile duct dilatation was noted on MRIs after 4 (2.8%) TACE sessions without any evidence of immediate clinical impact, whereas no portal vein thrombosis or biloma/liver infarct was observed.

### 3.4. Tumor Response

**Figure 1:**
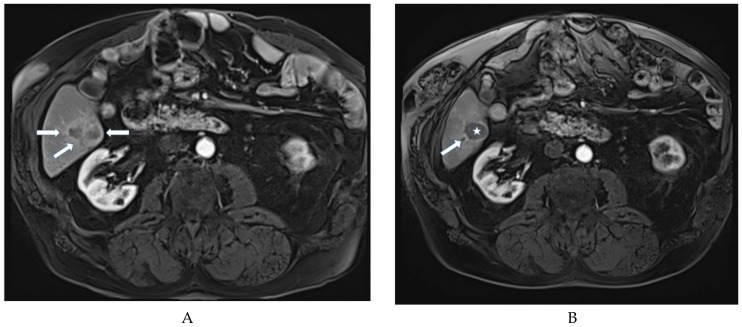
(**A**) Liver MRI (arterial phase) showing a 4-cm HCC (arrows) in segment VI in a 71-year-old cirrhotic patient. (**B**) MRI (arterial phase) performed three months after a super-selective DEB-TACE session (using 10 mg idarubicin loaded in 75-µm TANDEM beads administered through the segment VI artery) showing complete response (star) upon mRECIST, with dilatation of the segmental bile duct (arrow).

Assessments of tumor response after each TACE session are summarized in Table 3 ORR was observed in 61% after the first session (Figure 1), 64% after the second, 61% after the third, 50% after the forth and in 0% of cases after the fifth TACE session. DCR was observed in 73% after the first session, 75% after the second, 78% after the third, 100% after the forth and 50% after the fifth TACE session. Overall, the best ORR was 65%.

### 3.5. Outcome and Survival

**Figure 2:**
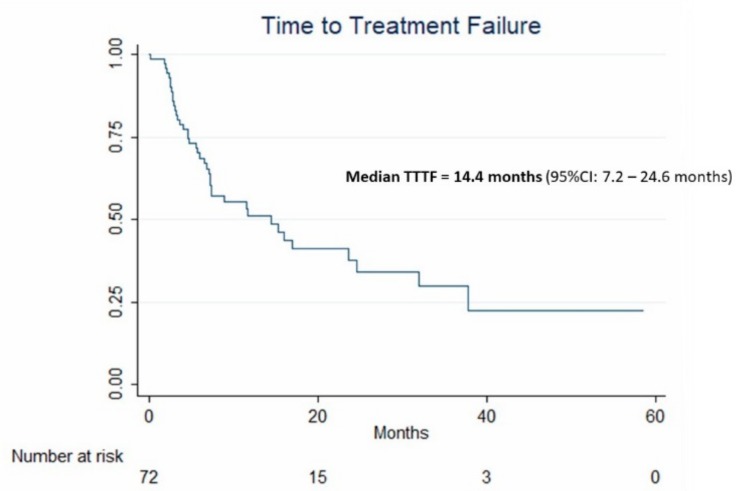
Kaplan–Meier curve and median 95% CI estimates of time-to-treatment failure (TTTF) in the 72 patients treated by idarubicin-loaded TANDEM.

Median follow-up lasted 14.3 months (95% CI: 11.2–18.8 months). Forty patients experienced treatment failure during follow-up, determining a median TTTF of 14.4 months (95% CI: 7.2–24.6 months) (Figure 2). Regarding treatment applied after TACE sessions, eight patients (11.1%) were downstaged to curative treatment: liver transplantation (*n* = 3), liver resection (*n* = 1), percutaneous ablation (*n* = 3), stereotactic body radiation therapy (*n* = 1). No patient was treated with conventional TACE after TANDEM-DEE. Second-line palliative treatments were selective internal radiation therapy (*n* = 5) and sorafenib (*n* = 19).

**Figure 3:**
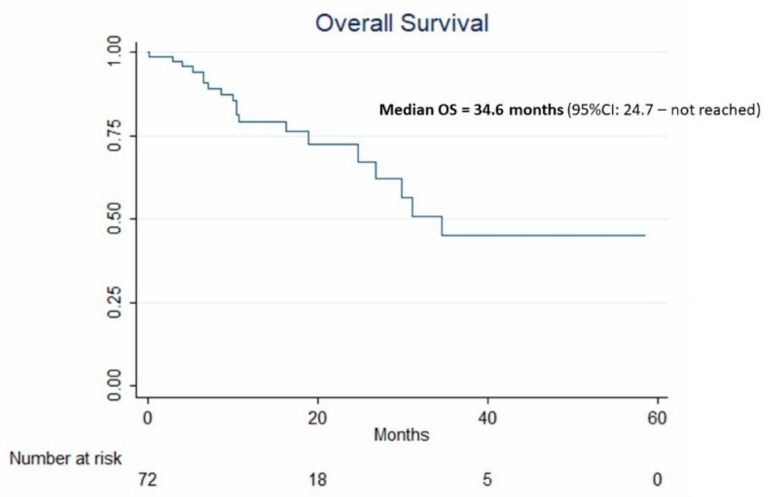
Kaplan–Meier curve and median 95% CI estimates of overall survival (OS) in the 72 patients treated by idarubicin-loaded TANDEM.

During follow-up, 19 patients died. Median OS was 34.6 months (95% CI: 24.7—not reached) (Figure 3).

## 4. Discussion

The efficacy of a TACE regimen based on idarubicin-loaded TANDEM seems promising. Indeed, we reported a median OS greater than the 30 months usually expected from TACE in the most recent guidelines [1]. However, OS captures survival resulting not only from TACE but also from additional treatment lines. TTTF as well as time-to-unTACEable progression (TTUP), which was used in the SPACE trial (i.e., one of the largest trial on doxorubicin-eluting beads so far) are interesting endpoints assessing the time during which the patient’s disease is controlled by TACE only. TTTF differs from TTUP mainly in that it takes into account any reasons for TACE discontinuation (including death) whereas, for TTUP calculation, death is censored. In our series, median TTTF was 14.4 months, whereas in SPACE, TTUP was 224 days (i.e., 7.5 months) for doxorubicin-eluting beads with a placebo arm [12]. In addition, the best ORR reported in our series (65%) is high compared with ORRs reported in the largest doxorubicin DEB-TACE trials: 51.6% in PRECISION V [4], and 28.1% in SPACE (doxorubicin DEB-TACE with a placebo arm) [12].

The results we report with idarubicin-loaded TANDEM might be explained by several factors: first, idarubicin has been shown to be the most cytotoxic drug among 11 anticancer agents (including doxorubicin, epirubicin and platinum derivatives) on three HCC cell lines [7]. The greater cytotoxicity of idarubicin is explained both by its greater penetration through the double-layer of tumor cells (owing to its greater lipophilicity) [13], and by its ability to overcome the multidrug resistance system [14], which is very active in HCC cells. Second, we used small and tightly calibrated microspheres (75–100 µm), which could have improved the results. Indeed, intra-tumoral vessels are smaller 300 µm [15]. A small size of drug-eluting embolics allows not only for a more distal embolization, but also for a greater drug concentration in the arterial network of the tumor [16,17], with improved drug coverage owing to the greater density of beads [18]. In terms of safety, adverse events were less frequent (*p* = 0.02) with small embolics (16% for 100–300 µm, 25% for 300–500 µm and 33% for 500–700 µm) in five prospective doxorubicin-eluting embolic TACE studies [19]. Even survival was shown to be better in patients treated with 100–300 µm drug-eluting embolics as compared to larger ones [20]. Third, with 88% unilobar disease and three or fewer HCC nodules in 92% of cases, our patients were highly selected. In the PRECISION ITALIA prospective trial [3], impressive results were reported with a 88.7% ORR, but in even more carefully selected patients (46.1% vs 15% BCLC A and a 3.1- vs. 5.5-cm mean diameter of the largest tumor in the PRECISION ITALIA trial vs in our study, respectively). In our series, only 15% of BCLC A patients were included, because our multidisciplinary tumor board tended to follow BCLC recommendations and keep ablations especially for tumors < 3 cm. Obviously, BCLC A patients have a better prognosis than BCLC B patients, which might have influenced the results of PRECISION ITALIA. Recently, the MIRACLE 1 multicentric trial reported a 67% ORR following doxorubicin-TANDEM DEB-TACE in very highlyselected patients as well (unilobar disease in 92% patients) [21]. In our practice, we made the choice to use DEEs in patients with limited tumor burden since a limited number of nodules makes super-selective treatment possible by catheterizing each tumor feeder sequentially. Conversely, in patients with numerous nodules, this approach is generally both time-consuming and useless. Super-selective TACE sessions not only improve response rates [22], but also limit the risk of toxicity. Indeed, we report here only 2.8% of liver/biliary injuries, whereas they have been reported after 35.7–36.8% of DEB-TACE sessions in previous studies [23,24]. Selectivity together with the use of small and tightly calibrated DEEs favors the deposition of microspheres within the tumor and, consequently, reduces the risk of damage to the portal triad when DEEs remain in non-tumoral arteries. Vesicant properties of anthracyclins are well known, and no difference is expected between idarubicin and doxorubicin. However, the greater the drug dose, the higher the risk of biliary damage [24,25]. Owing to its greater efficacy, idarubicin could be used at a much lower dosage (10 mg) [10] than doxorubicin (50–150 mg), which might have contributed to reduce the risk of biliary damage.

Several limitations of this study must be acknowledged, including the retrospective design and single-center setting, as well as the absence of a control group. Finally, since our policy was to favor patients with unilobar disease and three or fewer HCC nodules for DEB-TACE, our results cannot be attributed solely to the drugs used (i.e., idarubicin) or drug-eluting technology (i.e., TANDEM).

## 5. Conclusions

In conclusion, idarubicin-loaded TANDEM in selected patients with HCC is safe and results in high ORR, long TTTF and OS. This TACE regimen should be explored in future trials.

## Figures and Tables

**Table 1:** Baseline characteristics of patients and tumors.

	Number	%
Median Age, Range (Years)	71, 58–94	
Male/Female	66/6	92/8
Liver Cirrhosis (Yes/No)	64/8	89/11
Etiology (Alcohol/Virus/NASH/Others)	45/7/8/4	70/11/13/6
WHO Performance Status (0/1)	58/14	81/19
BCLC Stage (A/B)	11/61	15/85
Child-Pugh Class (A/B7)	65/7	90/10
Previous Treatment of HCC (Yes/No)	5/67	7/93
Unilobar/Bilobar Disease	63/9	88/12
No. of Nodules (1/2–3/>3)	48/18/6	67/25/8
Median Diameter of Largest Nodule, Range (mm)	55, 13–150	
Median Serum AFP, ng/mL (Range)	10, 3–17,660	
Median Serum PT, % (Range)	83 (41–100)	
Median Serum Bili, µmol/L (Range)	14 (4–48)	

**Table 2:** Grade 3–4 toxicity (*n* = 141).

Grade 3–4 Adverse Event (AE)	Number (%)
Any	73 (52%)
**Biological Disorders**	
Elevated alkaline phosphatase	2 (2%)
Elevated alanine aminotransferase	29 (21%)
Elevated aspartate aminotransferase	45 (32%)
Elevated γ-glutamyltranspeptidase	4 (3%)
Elevated lipase	1 (1%)
Hyperbilirubinemia	12 (9%)
Hyperglycemia	3 (2%)
**Clinical Disorders**	
Abdominal pain	6 (4%)
Fatigue	8 (6%)
Fever	4 (3%)
Ascites	1 (1%)
**Hepatobiliary Disorders**	
Gallbladder necrosis	1 (1%)
Liver failure	1 (1%)

**Table 3:** Tumor response, objective response rate and disease control rate.

Response (mRECIST)	After Session 1 (*n* = 72)	After Session 2 (*n* = 36)	After Session 3 (*n* = 22)	After Session 4 (*n* = 9)	After Session 5 (*n* = 2)
CR	10	6	2	1	0
PR	26	12	9	1	0
SD	7	3	3	2	1
PD	16	7	4	0	1
NA	13	8	4	5	0
ORR	61%	64%	61%	50%	0%
DCR	73%	75%	78%	100%	50%

CR: complete response; PR: partial response; SD: stable disease; PD: progressive disease; NA: not assessed; ORR: Objective response rate; DCR: Disease control rate.
